# Vaccination and COVID-19: beliefs and perceptions

**DOI:** 10.1192/j.eurpsy.2022.1347

**Published:** 2022-09-01

**Authors:** S. Stati

**Affiliations:** Hopital Arrazi De Sale, Psychiatrie, sale, Morocco

**Keywords:** belief, Perception, vaccine covid-19, Morocco

## Abstract

**Introduction:**

The COVID-19 pandemic has had a huge impact on societies, with hopes of a return to normalcy pinned on the availability of a COVID-19 vaccine. The success of a vaccination programme will depend on the participation rate among the population which is influenced by perceptions and attitudes that are partly determined by contextual factors

**Objectives:**

to study the associations between vaccination intention and theoretical background, contextual and socio-demographic factors in a demographic representation

**Methods:**

A cross-sectional, descriptive and analytical study was conducted from 3 December 2020 to 10 March 2021, using a questionnaire exploring demographics, psychiatric impact of the pandemic, general opinion of the pandemic and the vaccine, main reasons for being for or against the vaccine, and people’s affinity for the different vaccine currently on the market worldwide.

**Results:**

182 responses were collected, of which 83.5% were female, 50.5% were between 18 and 30 years of age, gender, contextual factors on vaccination uptake and type of vaccine showed a statistically significant difference with a P<0.005, between the 2 groups who agreed or disagreed with the vaccine uptake Univariate logistic regression analysis showed that female gender (OR = 0.193; 95% CI: 0.0437 -0.851) was independently associated with vaccine acceptance.

**Conclusions:**

The exploration of perceptions and beliefs concluded that there is an undeniable impact of contextual factors on the practice and acceptance of covid 19 vaccination among the general population in Morocco, and that awareness and psycho-education of the population is therefore desirable.

**Disclosure:**

No significant relationships.

